# Temporally selective contextual encoding in the dentate gyrus of the hippocampus

**DOI:** 10.1038/ncomms4181

**Published:** 2014-02-12

**Authors:** L.M. Rangel, A.S. Alexander, J.B. Aimone, J. Wiles, F.H. Gage, A.A. Chiba, L.K. Quinn

**Affiliations:** 1 Program in Neurosciences, University of California, San Diego, California, USA; 2 Salk Institute for Biological Sciences, La Jolla, California 92037, USA; 3 Department of Cognitive Science, University of California, San Diego, California 92093, USA; 4 School of Information Technology and Electrical Engineering, University of Queensland, Brisbane, Queensland 4072, Australia; 5 Present address: Department of Mathematics and Statistics, Boston University, Boston, Massachusetts 02215, USA; 6 Present address: Cognitive Modeling Department, Sandia National Laboratories, Albuquerque, New Mexico 87185, USA

## Abstract

A recent model of the hippocampus predicts that the unique properties of the dentate gyrus allow for temporal separation of events. This temporal separation is accomplished in part through the continual generation of new neurons, which, due to a transient window of hyperexcitability, could allow for preferential encoding of information present during their development. Here we obtain *in vivo* electrophysiological recordings and identify a cell population exhibiting activity that is selective to single contexts when rats experience a long temporal separation between context exposures during training. This selectivity is attenuated as the temporal separation between context exposures is shortened and is further attenuated when neurogenesis is reduced. Our data reveal the existence of a temporal orthogonalizing neuronal code within the dentate gyrus, a hallmark feature of episodic memory.

## Abstract (web summary)

The dentate gyrus of the hippocampus is required for processing the temporal context of memories. Here, Rangel *et al.* identify neurons in the rat dentate gyrus that also process the temporal separation between multiple experiences.

## Graphical abstract

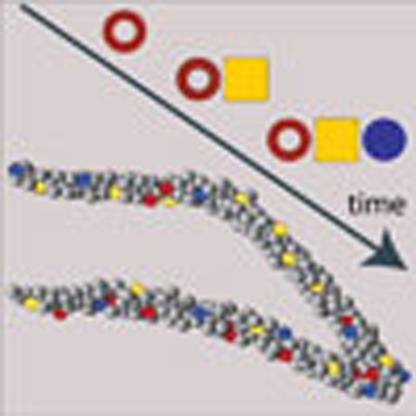

The brain has multiple memory systems, but the neural code underpinning our ability to remember when things happened remains elusive. The hippocampus is a neural structure subserving numerous functions, including the encoding and differentiating of episodic memories associated with the exploration of environments^1^,^2^,^3^. Consistent with this function in encoding spatial context, the dentate gyrus (DG) subregion of the hippocampus has been shown to be particularly important for discriminating between features such as spatial locations or environmental cues^4^,^5^.

Episodic memories are also encoded according to the temporal contexts in which they occur. It is possible for one to remember that two different experiences in the same environment occurred at different times. For example, one can distinguish whether two different exercise courses in the same school gymnasium occurred on the same or different weeks. The temporal context of an event on the timescale of weeks appears to be relevant to effective learning and DG function in rats^6^,^7^. However, the mechanisms through which such temporal contextual information is encoded in DG activity and how it relates to the hippocampal code for spatial context remain to be determined.

As one of the only brain regions to exhibit adult neurogenesis or the continual birth of new neurons throughout life^8^, the DG may be uniquely adapted to mediate encoding of temporal context over these longer time frames. Immature adult-born neurons in the DG exhibit characteristic stages of development that include a transient period (approximately 1.5–6 weeks) of both intrinsic and synaptic hyperexcitability^9^,^10^,^11^. Based on these features, computational models suggest that episodes occurring closer in time to each other should elicit activity from a common set of immature cells due to their enhanced excitability. This period of enhanced excitability would provide a similarity in DG output for temporally proximal episodes that does not exist for episodes separated further in time, that is, a temporal integration of inputs^12^,^13^.

A potential outcome of such temporal integration could be the creation of long-term temporal separation of experiences through the recruitment of distinct cell populations. Whereas events separated by short timescales are expected to be encoded by the same population of immature adult-born cells, events separated by long timescales are predicted to recruit unique populations of mature adult-born cells.

To test the temporal separation hypothesis, two distinct sets of experiments were performed. The first tested the general prediction that a set of neurons should exist that selectively, temporally encodes environments/experiences separated by long periods of time (>3 weeks). A large proportion of cells demonstrated activity selective to a single environment when introductions to the different environments were separated by intervals >3 weeks. The second set of experiments tested the effect of reducing temporal separation upon the extent of context selectivity and the effect of adult neurogenesis knockdown on such selectivity. The proportions of cells with activity selective to single environments were reduced with shortened timelines, and further reduced with neurogenesis knockdown. The results point to a role for the DG in the temporal parsing of events required for episodic memory.

## Results

### Long temporal separation experiment

**Figure 1:**
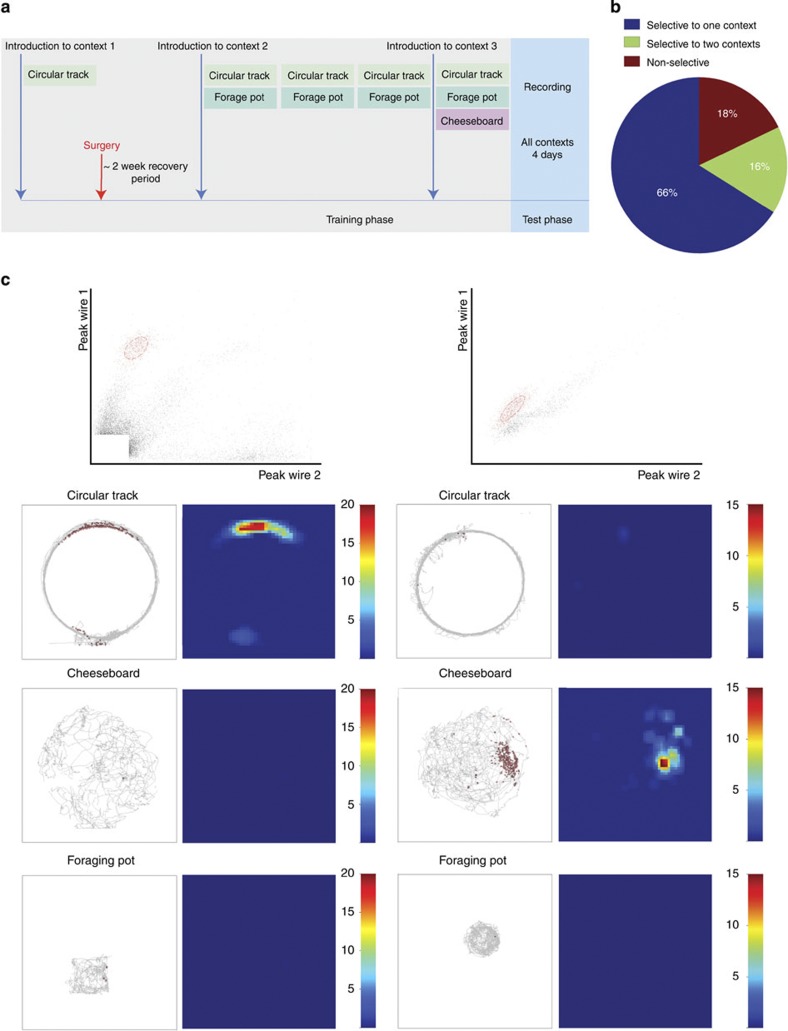
Single granule cells preferentially exhibit selective contextual encoding in the 3-week separation group. (**a**) Example timeline of context exposures (coloured boxes=1 week). (**b**) The percentage of place cells (*N*=56) selective to one context (blue), two contexts (green) or exhibiting activity in all three contexts (red) using a place field criterion. (**c**) Projections of spike amplitude across recording wires from two representative cells (upper). Rat paths within each context (grey) superimposed with spike locations (red dots) shown to the left of firing rate maps for each of the three contexts. Colour bar indicates the frequency of spikes (Hz) for a given pixel.

**Figure 2:**
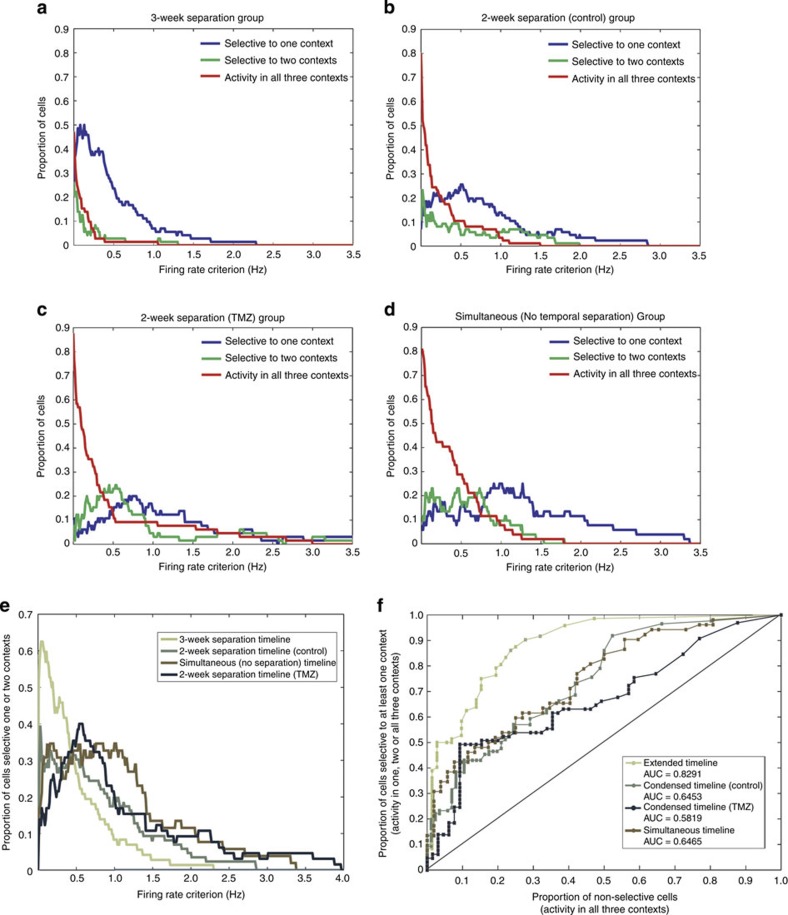
Analysis of selectivity using firing rate criteria. (**a**–**d**) The proportion of cells with activity selective to one (blue), two (green) or all three (red) behavioural contexts for 200 firing rate thresholds between 0.01 and 3.5 Hz. (**e**) The proportion of cells with activity selective to one or two contexts with increasing firing rate threshold for all groups. (**f**) Receiver-operating characteristic curves for each group of the proportion of non-selective cells plotted against the proportion of cells selective to at least one context with increasing firing rate criteria from 0.01–10 Hz (right to left).

In the first experiment, recordings of DG neurons were obtained over 4 days of re-exposure to three distinct contexts separated in time during training (Fig. 1a). To ensure the recruitment of different populations of neurons in each context while minimizing age-related reductions in adult neurogenesis, initial experiences in each context were separated by intervals greater than, but approximately, 3 weeks. Additionally, the different environments/experiences occurred within the same global space, not in separate rooms, where there has been shown to be a lack of recruitment of distinct populations^14^. Introductions to novel contexts were paired with re-exposure to previously introduced contexts to re-engage memory of remote experiences and encourage rats to discriminate between the distinct behaviour requirements of each. Seventy-two putative granule cells were recorded from three rats (See Methods for characteristics used for interneuron exclusion) in the first experiment. We characterized the nature of context selectivity in these cells by using a place field criterion (see Methods for place field detection method). Fifty-six cells met a place field criterion in at least one context. Of these 56 cells, 66% exhibited place fields selective to one context, whereas 16% exhibited fields in two contexts and 18% exhibited fields in all three contexts (Fig. 1b). We also utilized a firing rate criterion to determine whether a cell was active in a single context. Since the mean firing rate cutoff for ‘active’ is arbitrary, we looked at a continuum of firing rates and found a high amount of selectivity to a single environment using previously utilized cutoffs (0.1 and 0.25 Hz, Fig. 2a, Supplementary Table 1)^15^. The same was observed for a wide range of cutoffs. Though it is unclear which measure best quantifies the most meaningful form of activity for downstream targets, both measures reveal a selectivity of firing for single contexts when the introduction to each context is separated by long temporal intervals. We chose to focus primarily on the place field criterion, however, because this measure is readily relatable to the known functions of other hippocampal regions.

The large percentage of cells selective to one context in the first experiment indicates that the DG does, in fact, selectively encode environments that are temporally distinct on the 3-week time scale.

### Reduced timeline and neurogenesis knockdown experiment

**Figure 3:**
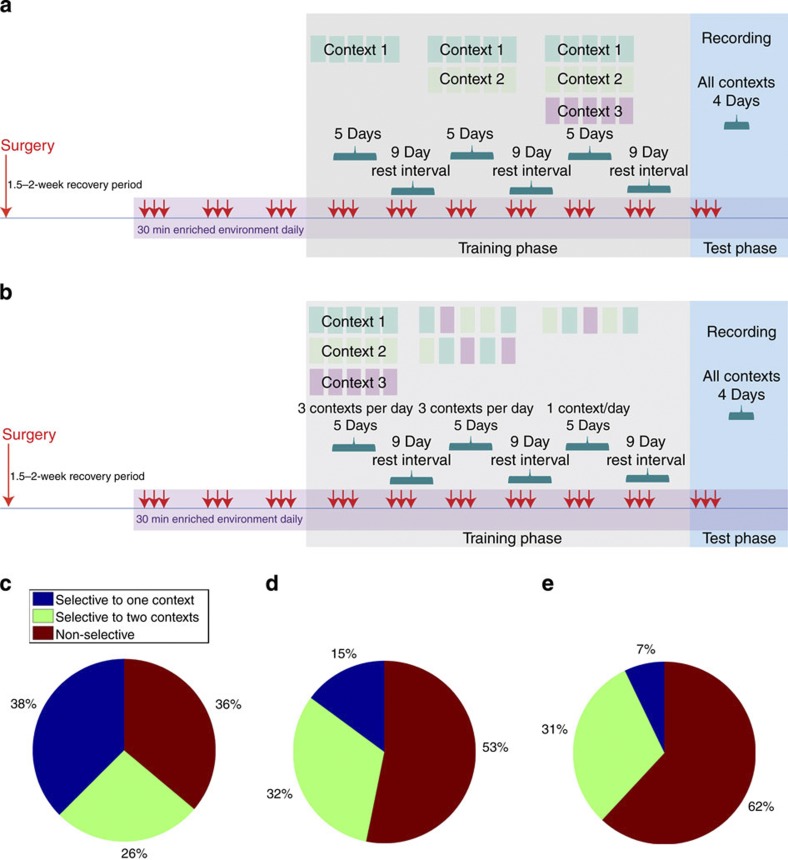
Systematic reduction in context selectivity using a place field criterion. (**a**) Timeline of context exposures for the 2-week separation group. Red arrows indicate days in which TMZ or saline injections were given. (**b**) Timeline of context exposure for the simultaneous (no separation) group. The percentage of cells in the 2-week separation control group (*N*=72; **c**), simultaneous group (*N*=47; **d**) or 2-week separation TMZ-treated group (*N*=42; **e**) selective to one context (blue), two contexts (green) or exhibiting activity in all three contexts (red) using a place field criterion.

In the second experiment, the temporal influence upon context selectivity and the effect of neurogenesis knockdown upon such selectivity were tested. A 2-week interval (the 2-week separation timeline) was chosen to test whether a shorter interval would still result in cells selective to different contexts as well as to maximize the extent of neurogenesis knockdown during context exposure, given the timeline of efficacy for our chosen knockdown procedure (Fig. 3a). While current recording techniques do not permit identification of context-selective cells as either adult or immature DG granule neurons, it is nevertheless possible to determine the impact of selective reduction of neurogenesis on the degree to which context selectivity in DG neuronal activity actually occurs. Thus, we sought to determine whether the reduction of adult neurogenesis impacted context selectivity in animals experiencing the 2-week timeline. Additionally, we compared context selectivity when initial encounters with the same three environments all occurred on the same day (the simultaneous exposure timeline; Fig. 3b) To enhance the survival of adult-born neurons and their potential contribution to DG function, rats were given 30 min of enriched environment exposure daily throughout the course of the experiment^16^.

In the 2-week separation timeline, 94 cells were recorded from seven rats, with 72 cells meeting a place field criterion in at least one context (Fig. 3c, for representative spike locations and firing rate maps see Supplementary Fig. 1, for results by individual rat see Supplementary Fig. 2). Of these, 38% exhibited place fields in only one context, whereas 26% exhibited fields in two contexts and 36% exhibited fields in all three contexts. Fifty-two cells were recorded from five rats exposed to the simultaneous timeline. Of these cells, 47 met the place field criterion in at least one context, with 15% exhibiting place fields selective to one context, 32% exhibiting fields in two contexts and 53% exhibiting fields in all three contexts (Fig. 3d, for representative spike locations and firing rate maps see Supplementary Fig. 1, simultaneous versus 2-week timeline: *χ*^2^=7.33, d.f.=2, *P*=0.026).

Context selectivity was found in the 2-week separation group; however, it was reduced relative to animals undergoing the 3-week separation timeline (3-week timeline versus 2-week timeline: *χ*^2^=10.41, d.f.=2, *P*=0.005). Context selectivity was further reduced in animals experiencing the simultaneous timeline, demonstrating that the context selectivity observed in the first experiment was indeed attributable to temporal differences in the times of initial encounter with the environments.

Having found that selectivity to contexts separated by 2 weeks still occurs, albeit to a lesser degree than in the 3-week separation timeline, we then assessed the role of adult-born neurons in selective encoding at the 2-week time scale. An anti-mitotic chemotherapeutic agent, temozolomide (TMZ), was used to transiently knock down levels of adult neurogenesis^17^. We determined that three injections of 12.5 mg kg^−1^ TMZ each week produced ~40% neurogenesis knockdown after 2 weeks, which was fully recovered by 4 weeks, despite weekly injections throughout the course of the experiment (Supplementary Fig. 3). This dosage and injection regime resulted in a similar knockdown of adult neurogenesis to what has been reported previously^18^. Earlier studies have also indicated no detriments in overall cognitive ability or memory for previously learned associations due to long-term TMZ drug treatment^19^. Adult-born neurons have been shown to demonstrate action potentials as early as 1.5 weeks but remain more plastic than their mature counterparts until 6 weeks^16^,^20^. We predicted that if TMZ injections were administered 3 weeks prior to context exposures in the 2-week separation group, a transient neurogenesis knockdown would reduce context selectivity for the first two contexts but that this selectivity could recover by the third context presented due to the greater availability of adult-born neurons (Fig. 3a).

Sixty-five cells were recorded from six rats receiving TMZ and exposed to the 2-week separation timeline. Forty-two cells from this group met the place field criterion in at least one context. Of these 42 cells, 7% exhibited place fields selective to one context, whereas 31% exhibited fields in two contexts and 62% exhibited fields in all three contexts (Fig. 3e, for representative spike locations and firing rate maps see Supplementary Fig. 1). This distribution was significantly different from that of cells in the 2-week separation timeline that did not receive TMZ (Fig. 3c, control versus TMZ: *χ*^2^=13.36, d.f.=2, *P*=0.001), but it was not significantly different from that of cells in the simultaneous timeline group (Fig. 3d).

These results indicate that reduction of neurogenesis prior to initial environment encounters under the 2-week separation timeline reduces context selectivity during subsequent re-exposure to the environments.

### *I*_Context_ analysis

**Figure 4:**
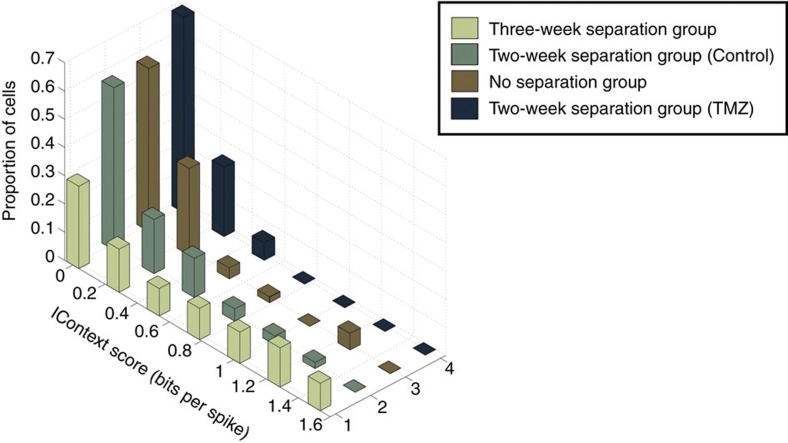
Reductions in context selectivity using *I*_Context_ reflect the nature of the training paradigms. The proportion of cells with *I*_Context_ scores between 0 and 1.6 bits per spike in the 3-week separation group (*N*=72, light green), 2-week separation control group (*N*=94, dark green), simultaneous (*N*=52, no separation) timeline (brown) group and 2-week separation TMZ-treated group (*N*=65, dark blue). The extended timeline group has the largest proportion of cells with high *I*_Context_ scores.

To quantify the extent to which cell activity could be predictive of the context the rat was in, we designed a contextual information score (see Methods, Fig. 4). Rats exposed to the original 3-week separation timeline had a greater proportion of cells with higher contextual information than rats exposed to the 2-week separation or simultaneous timelines (*N*_3-week_=72 cells, *N*_2-week/control_=94 cells, Wilcoxon rank sum *P*=7.76 × 10^−7^, Kolmogorov–Smirnov *P*=6.15 × 10^−6^ and *N*_simultaneous_=52 cells, Wilcoxon rank sum *P*=3.83 × 10^−6^, Kolmogorov–Smirnov *P*=4.95 × 10^−6^, respectively). Control rats exposed to the 2-week separation timeline had a greater proportion of cells with higher contextual information than rats exposed to the same timeline receiving TMZ (N_2-week/TMZ_=65 cells Wilcoxon rank sum *P*=0.02, Kolmogorov–Smirnov *P*=0.01). There was also a significant difference between TMZ rats exposed to the 2-week separation timeline and rats exposed to the simultaneous timeline (TMZ 2-week versus simultaneous: Wilcoxon rank sum *P*=0.128, Kolmogorov–Smirnov *P*=0.015).

However, there was no significant difference in contextual information between control rats exposed to the 2-week separation timeline and rats exposed to simultaneous timeline (control 2-week versus simultaneous: Wilcoxon rank sum *P*=0.53, Kolmogorov–Smirnov *P*=0.60). The fact that there is a distinction between these two groups using a place field criterion suggests that the two analytical measures are exposing distinct aspects of contextual encoding. Whereas the place field analysis assesses the selectivity of spatial encoding within each of the three contexts, the *I*_Context_ score measures selectivity based on firing rates, irrespective of spatially related activity.

## Discussion

Our studies reveal the existence of a neural code that separates events in time. Segregating exposure to contexts by >3 weeks results in a very large proportion of cells that are selective to a single context. The capacity of DG neurons to selectively encode temporally distinct contexts enables them to participate in the separation of episodic or contextual memories over long durations, extending the known ability of the hippocampus to encode both time and space^21^,^22^,^23^.

Our results support the hypothesis that the continual addition of neurons with temporally delineated periods of hyperexcitability permits the DG to specifically encode a single context if exposure to that context is temporally segregated from other exposures. Thus, space may be coded as a function of time by the periodic addition of new neurons into the hippocampal circuit. Under this hypothesis, the fact that longer intervals between contexts recruit more distinct populations of putative adult-born cells than shorter intervals suggests a crucial time point in the maturation of adult-born neurons for effective discrimination of temporal contexts. This time point is elucidated by the observed changes in selectivity between the 2-week and 3-week separation groups, and by the fact that rats with reduced neurogenesis (using TMZ) demonstrated a large proportion of cells selective to three contexts in similar proportions to the group that was trained without any temporal separation between contexts (Fig. 3d,e). The findings in the TMZ-treated and simultaneous exposure groups are consistent with previous DG studies conducted without significant temporal context separation^15^. These studies showed that both small and large changes across environments recruit activity from an overlapping, small population of DG cells (potentially mature or immature) that distinguish between environments through changes in firing rate rather than through global remapping.

Alternatively, the presence of immature adult-born cells could significantly impact the overall excitability of the mature cells in the region by driving a majority of the feedback inhibition onto the granule cell layer^24^,^25^,^26^. Consistent with this hypothesis, the neurogenesis knockdown group exhibited cells with overall higher mean firing rates than control rats given the same temporal separation between contexts (Supplementary Table 1). We do not believe that this increase in mean firing rate is due solely to TMZ administration, as similar differences in mean firing rate were observed between the 3-week, 2-week and no separation groups in which the only difference between the groups is the timing between initial context presentations. Nevertheless, it is possible that reduced temporal separation during training differentially affects the overall levels of excitability in the circuit during re-exposure in a manner that is similar to transiently reducing levels of adult neurogenesis during training. While the exact cellular mechanism of the observed context selectivity is unknown, the net effect is consistent with our conclusion that the timing of initial exposure is a critical determinant of DG activity.

Using temporal separation of context presentation, we revealed a mechanism by which the DG encodes contexts with unique, non-overlapping cell populations. This ability of the DG was theoretically predicted in models in which a small number of neurons within a relatively dense cell layer function as a mechanism to ‘pattern separate’ or ‘orthogonalize’ multiple experiences^27^,^28^. Our data confirm this prediction by revealing the existence of a temporal orthogonalizing neuronal code within the DG.

## Methods

### Firing rate and place field criterion analyses

The firing rate in each context was determined by dividing the total number of spikes per context by the time spent in the context (Supplementary Tables 1 and 2, Supplementary Fig. 4). To assess the presence of place field activity in each context, place field candidates were first identified that had a <3 Hz overall mean firing rate, place fields covering at least three 5 cm × 5 cm contiguous pixels before reducing to 10% of peak firing rate, and a peak pixel of at least 1 Hz that was also 2 s.t.d. above the mean firing rate of the cell in a given context^29^,^30^. To meet a place field criterion for a context, these candidates were then required to have a statistically significant spatial information score for that context when compared with 1,000 iterations of the same spike times for a given cell with randomized spatial position. The number of cells with place fields in one, two or all three behavioural contexts was used to assess the extent of context selectivity.

### Informational analysis of context selectivity

To assess selective activity to a given behavioural context, we developed a novel measure of contextual information using the following equation:


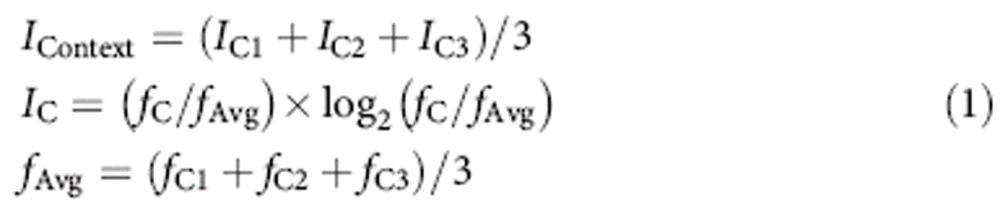


where *f*_C_ is the number of spikes occurring within a context divided by total time spent in a context, *f*_Avg_=the average firing rate across all contexts, *I*_C_=the information score of a given context in bits/spike and *I*_Context_=the amount of information present in each spike regarding contextual inhabitance.

### Behavioural contexts

The three behavioural contexts were a 13′′ × 13′′ foraging pot containing rat bedding and randomly placed ¼ pieces of Honey Nut Cheerios (for one rat, the foraging pot was a circular pot that had larger walls to prevent the rat from jumping over the edges), a 48′′ diameter circular track with a 3′′ wide pathway and either ¼ pieces of Honey Nut Cheerios or chocolate sprinkles in a reliably rewarded location that was shifted to a different track location up to three times per session and a 48′′ diameter circular cheeseboard with randomly placed chocolate sprinkles and three presentations of a large food reward (a 2′′ diameter dish filled with chocolate sprinkles) at random time intervals and random locations for a period of >30 s (Supplementary Fig. 5). All three behavioural contexts were presented in the same location of the same recording room containing constant external cues. The time length of exposure to each context differed between contexts to allow full exploration of the larger contexts and minimize oversampling of the smaller contexts. Exposure time lengths during both training and test sessions for the foraging pot, circular track, and circular cheeseboard were ~10, 15 and 20 min, respectively.

### Timeline for simultaneous and temporally distinct exposure

Rats were exposed to the three behavioural contexts according to one of three timelines: an extended 3-week separation timeline, in which the initial exposure to each context was separated by >3 weeks (Fig. 1a); a 2-week separation timeline, in which the initial exposure to each context was separated by 2 weeks (Fig. 3a) or a simultaneous timeline in which there was no temporal separation between initial context exposures (Fig. 3b). Temporal intervals of >3 weeks between behavioural context exposures in the extended timeline would ensure a length of time between contexts that was long enough to recruit different populations of adult-born neurons but short enough to prevent age-related reductions in adult neurogenesis proliferation. In the 3-week separation timeline (*N*=3 rats), rats were first trained to run around the circular track before undergoing surgery for microdrive implantation. After a recovery period of at least 2 weeks and during several weeks in which microdrive wires were slowly lowered to the granule cell layer of the DG, rats were re-trained on the circular track and began exposure to the foraging pot environment. During this time in training, they received exposure to both contexts each day. Once stable cell recording could be obtained from the granule cell layer and at least 3 weeks after initial exposure to the foraging pot, rats were exposed to the circular cheeseboard environment. During this time in training, they received exposure to all three contexts each day. All analyses of cell activity were restricted to days in which rats received exposure to all three contexts on the same day.

In the 2-week separation timeline (*N*=7 control rats, *N*=7 TMZ-treated rats), initial exposure to each behavioural context was separated by a fixed 2 weeks to provide an interval between contexts that was long enough to recruit a new population of adult-born neurons but short enough to allow for the fullest extent of knockdown during contextual exposure, given the timeline of efficacy for the chosen knockdown procedure (Fig. 3a). For the first context exposure, the rat was exposed to only a single behavioural context for 5 days followed by a 9-day rest period without exposures. In subsequent exposures to the second and third behavioural contexts, exposure to the new context also included exposure to previously presented contexts in the same recording day. Nine days after the end of training, rats received a 4-day test phase that included presentation of all three behavioural contexts. All analyses of cell activity were restricted to test days. The order of exposure to each of the three behavioural contexts was counterbalanced across groups.

In the simultaneous timeline (*N*=5 rats), rats were exposed to all three behavioural contexts in the same day for the first week of training. In subsequent exposure weeks, the numbers of exposures to each context were limited to two contexts per day and finally one context per day to equate time spent in each behavioural context across groups.

### Adult neurogenesis knockdown

TMZ is an anti-cell proliferation drug that has been shown to reduce cell proliferation in the DG by >80% in mice and cell survival in rats by >40%^17^,^19^. After testing several dosages of the drug in rats, we found the optimal dosage to be three i.p. injections of 12.5 mg kg^−1^ each week for 2 weeks. This injection regime produced a significant reduction in cell proliferation by ~40% after 2 weeks, with recovery of neurogenesis levels through consistent use of this injection regime after 4 weeks (Supplementary Fig. 3). To prevent possible side effects from stopping treatment after the beginning of the experiment, three injections of 12.5 mg kg^−1^ TMZ were performed every week for 10 weeks (beginning 3 weeks before the initial context exposure). Controls received saline injections of the same volume and frequency as TMZ-treated rats. There were no differences in exploratory behaviour between control and TMZ-treated groups (see further Methods for analysis of time spent in each context per group).

### Rats

All animal procedures were performed in accordance with NIH and local IACUC guidelines. Twenty adult, male Long-Evans rats were used as subjects. The rats were housed individually and maintained on a 12-h light/dark cycle. They were acclimated to the colony room for 3 days and handled daily for at least 2 weeks before beginning the experiment, during which time they were placed on food restriction until they reached 85–90% of *ad libitum* weight. Rats were 3 months old at the time of surgery. Weight ranged from 300 to 350 g. Water was available at all times. All behavioural testing occurred during the rats’ light cycle.

### Microdrive implantation surgery

A microdrive consisting of four tetrodes of 17-μm platinum iridium wire was surgically implanted using stereotaxic procedures (from bregma A/P: −4.0, M/L: +2.2 mm, D/V: −2.2 mm) and was lowered into the granule cell layer of the DG (D/V: ~2.7 mm) until the appearance of place cell single units, ‘dentate spikes’ and complex high-frequency (16–90 Hz) local field potential activity.

### Neural recordings

During targeting of the DG granule cell layer, each wire of the microelectrode bundle was first checked for neural activity. The quietest wire was found and served as a reference electrode. If no activity was found on the recording wires, the bundle was advanced in 10-μm increments until activity was found. Wires were not turned within 24 h before a recording session.

The activity on each wire was passed through a high-impedance OpAmp headstage that held a set of light-emitting diodes (Neuralynx Technologies, Bozeman, MT). A multiwire flexible cable connected the preamplifiers to a 32-channel commutator (Neuralynx Technologies). The single-unit signal was filtered at 600 Hz (low pass) to 6,000 Hz (high pass) and amplified through Neuralynx Lynx-8 differential programmable amplifiers. The local field potential signal was filtered at 1 Hz (low pass) to 475 Hz (high pass). The amplifiers were integrated with the Cheetah data acquisition program (Neuralynx Technologies), wherein the acquired analogue signals were digitally converted at a rate of 30,303.00 KHz (DT 2821 Data Translation, Marlboro, MA) prior to storage. The position of the rat was monitored by a set of light-emitting diodes placed on the headstage. A video tracking system (SA-2 Dragon Tracker, Boulder, CO) registered the position of the diodes with a sampling frequency of 30 Hz. All recording sessions began with 5 min of baseline recording activity in a home cage environment. Single cells were identified using Offline Sorter (Plexon, Dallas, TX) to compare relative amplitude of each spike across tetrode wires. Final microdrive wire location was verified post-mortem in 40-μm sections using a Nissl stain (Supplementary Fig. 6).

### Interneuron exclusion

To exclude interneurons from selectivity analyses, all cells that exhibited both a mean firing rate in any context above 5 Hz and a mean waveform width of <150 μs at half of mean peak amplitude were excluded. In general, putative granule cells discharged at low mean firing rates (<3 Hz with mean spike waveform widths of at least 0.15 ms taken at 50% of peak amplitude). In contrast, putative interneurons typically demonstrated mean peak firing rates of >5 Hz with mean spike waveform widths of <0.15 ms.

### Analysis of selectivity using firing rate criterion

To assess context selectivity using firing rates, the proportion of cells selective to one, two or all three behavioural contexts was calculated using a firing rate criterion. Since choosing a single firing rate criterion can be quite arbitrary, we examined context selectivity using a continuum of firing rate criteria from 0.01 to 3.5 Hz in increments of 0.01 Hz (Fig. 2a–e). To quantify differences in selectivity over multiple firing rate criteria across groups, a receiver-operating characteristic curve was made for the changing proportion of cells with activity in all three contexts compared to the proportion of cells selective to at least one context for each increase in the firing rate criterion in 0.01 Hz steps (Fig. 2f). The area under the curve for each group was calculated using trapezoidal approximations.

### Spatial informational analysis

A spatial information score was calculated for all cells in each behavioural context^31^,^32^. This spatial information score formula was calculated as follows:


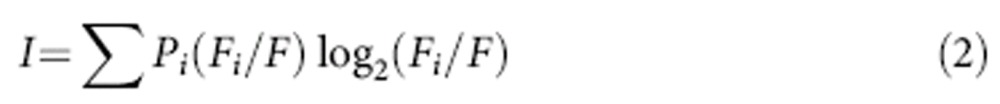


Where *i* is the pixel number, *P*_*i*_ is the probability of occupancy in pixel *I* and *F*_*i*_ is the mean firing rate for pixel *i*.

*F* is the overall mean firing rate of the cell.

### Burst index

A burst index was calculated for both single-fielded and multi-fielded cells (Supplementary Table 3), which divided the number of times two consecutive spikes occurred within 6 ms by the total number of interspike intervals during the course of a recording session^29^.

### Analysis of time spent in each context per group

To ensure that any differences in context selectivity across groups was not attributable to differences in rat mobility or time spent in select areas of a context (such as a rat sitting in a cell’s place field), an immobility bias was calculated for each cell. The correlation between a cell’s mean firing rate in a pixel and the time spent in each pixel was first calculated for each context. These correlations were then added together to determine any firing rate biases due to immobility. If one experimental group exhibited higher immobility biases than another, then differences in rat activity levels could feasibly account for changes in cell selectivity. There were no significant differences in immobility biases across groups.

### Analysis of place field representation biases across contexts

The numbers of fields in each context for each group were calculated and compared to observe whether place field overrepresentation of one context could drive the place field selectivity observed in this study (Supplementary Table 4). While there was a disproportional representation of the three different contexts, these proportions were not significantly different across groups.

### Immunohistochemistry

To quantify levels of neurogenesis in pilot rats receiving TMZ for 2 or 4 weeks, rats were injected with 50 mg kg^−1^ 5-bromo-2′-deoxyuridine (BrdU) for 4 consecutive days during week 2 or 4 of TMZ treatment, respectively (Supplementary Fig. 3). This measure indicates the number of dividing cells after 2 and 4 weeks of TMZ administration. After 24 h from the final injection of BrdU in each group, rats were perfused with 0.1 M PO_4_ followed by 4% paraformaldehyde and rat brains were removed from the skull to be incubated for at least 48 h in 30% sucrose at 4 °C. Frozen brains were then cut into 40-μm coronal sections with a microtome. Sections from every 240 μm spanning the whole DG were selected (1 of every 6 sections). Sections were post-fixed for 50 min in 4% paraformaldehyde at room temperature and incubated for 30 min in 2 N HCl at 37 °C followed by 1 N Borate Buffer at room temperature. Sections were then incubated at 4 °C in primary Rat anti-BrdU (1:100) for 72 h (Catalogue no. OBT0030, Accurate, NY). Finally, they were incubated at room temperature in secondary Donkey anti-Rat IgG Dylite 488 (1:250) for 2 h (Catalogue # 712-485-153, Jackson ImmunoResearch, PA). Every BrdU-labelled cell was counted at × 40 magnification in 10 consecutive 40-μm sections spaced 240 μm apart. These counts were then used to estimate the total number of BrdU-labelled cells in each dorsal DG. Two different experimenters who were blind to rat identity counted cells independently to ensure that cell counts were consistent across experimenters. In half of the rats, total numbers were additionally verified using unbiased stereological technique (StereoInvestigator, MBF Bioscience, CA).

## Author contributions

L.M.R. and A.S.A. performed TMZ drug preparations and injections; L.M.R. and A.S.A. performed histology; L.M.R., A.S.A. and L.K.Q. performed microdrive implant surgeries; L.M.R., A.S.A. and L.K.Q. performed behavioural experiments and electrophysiological recordings; L.M.R. and J.B.A. performed analyses; L.M.R., L.K.Q., J.B.A., J.W., A.A.C. and F.H.G. designed the study; A.A.C. and L.K.Q. provided oversight of experiments; L.M.R and L.K.Q wrote the paper. All authors discussed the results and commented on the manuscript. Stereological counts were performed in F.H.G.’s laboratory. All behavioural recording studies were performed in A.A.C.’s laboratory.

## Additional information

**How to cite this article:** Rangel, L. M. *et al.* Temporally selective contextual encoding in the dentate gyrus of the hippocampus. *Nat. Commun.* 5:3181 doi: 10.1038/ncomms4181 (2014).

## Supplementary Material

Supplementary Information Supplementary Figures 1-6 and Supplementary Tables 1-4
