# Supplementary material for: Temporally selective contextual encoding in the dentate gyrus of the hippocampus
Source: Nat Commun. 2014 Feb 12;5:3181. doi: 10.1038/ncomms4181 (PMC3929785; doi:10.1038/ncomms4181)
Supplement: Supplementary Information — Supplementary Figures 1-6 and Supplementary Tables 1-4 [file ncomms4181-s1.pdf]

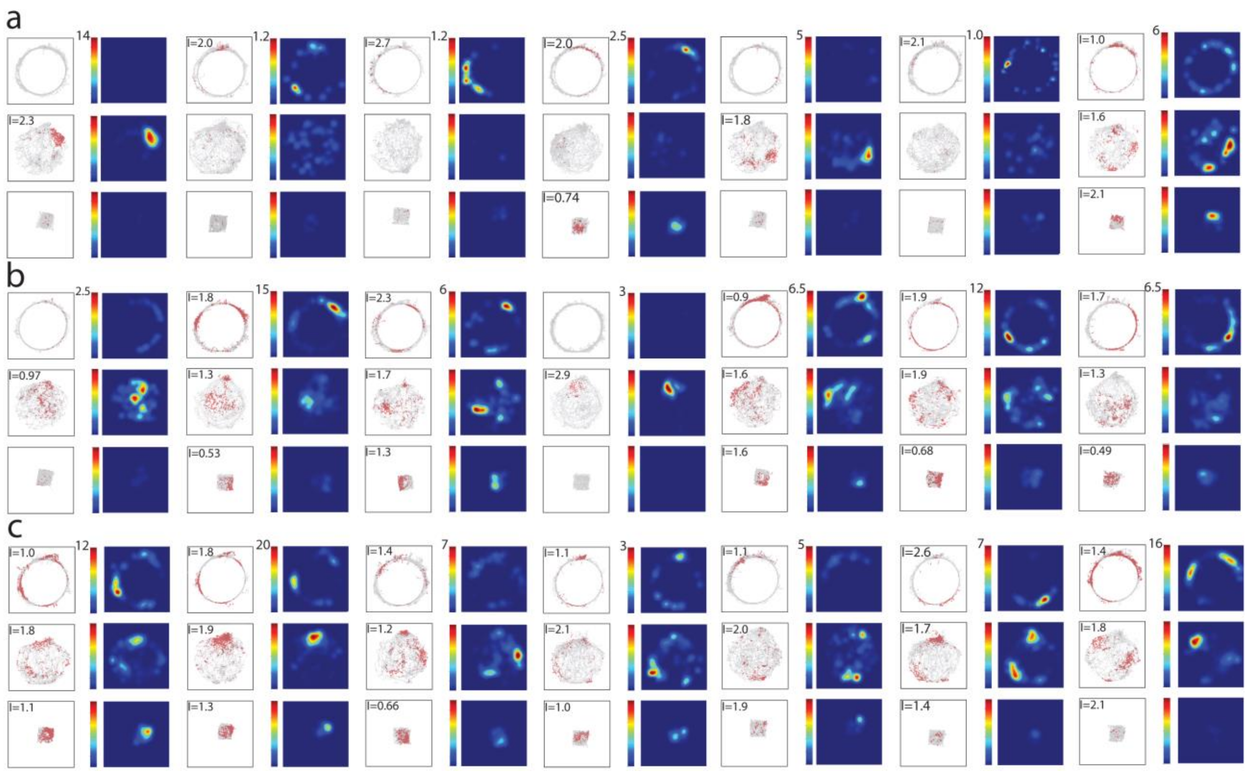

Supplementary Figure S1: Seven representative cells from the 2-week separation (Control and TMZ) and simultaneous context groups. Seven sample cells from the (**a**) 2-week separation (control) group, (**b**) simultaneous (no separation) group, and (**c**) the 2-week separation (TMZ) group. Rat paths within each context (gray) with superimposed spike locations (red dots) are shown to the left of rate maps for each of the three contexts (arranged from top to bottom to show circular track, circular cheeseboard, and forage pot exposures, respectively). Color bars indicate the peak mean firing rate calculated within a single pixel (Hz) for a given cell across all three contexts. Color bar scales are matched across all three contexts for each example cell. For cells that met a place criterion for a given context, the spatial information score is indicated in the upper right corner of spike location figures.

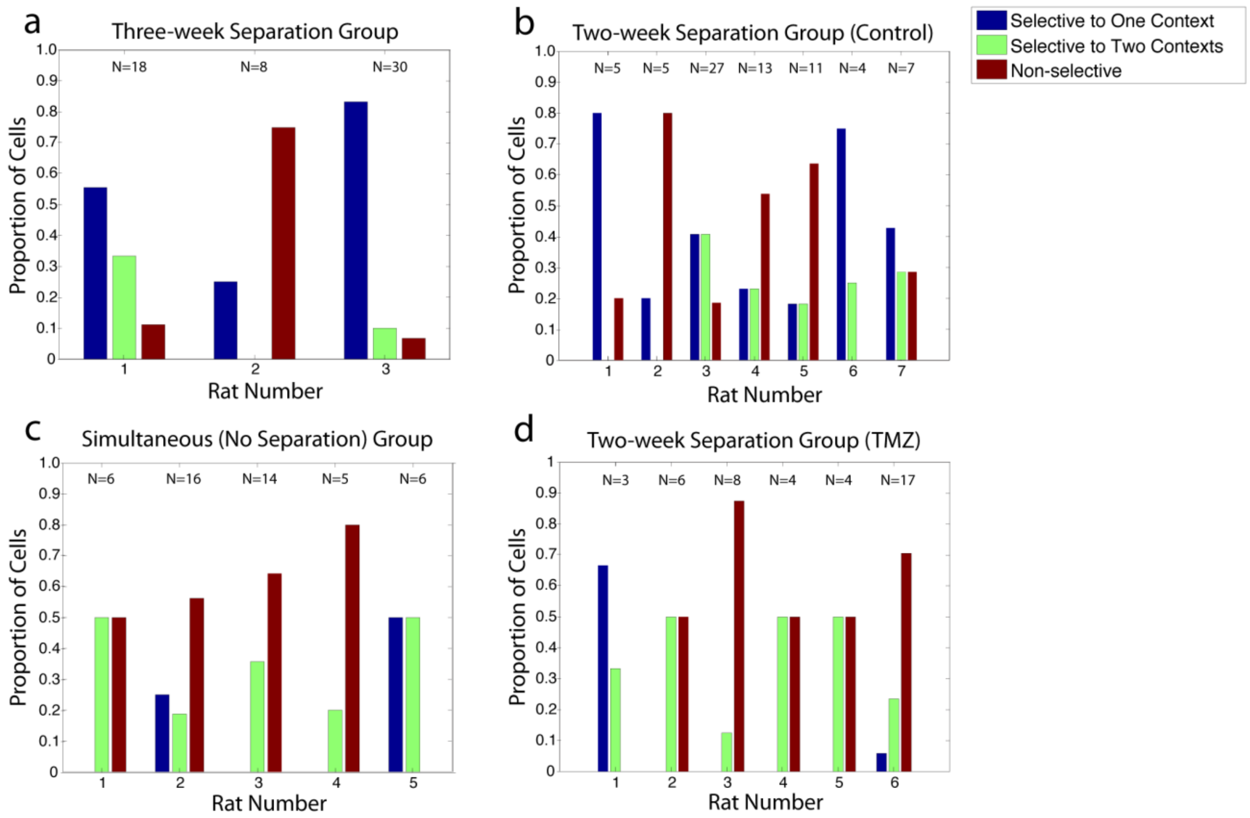

Supplementary Figure S2: Contextual selectivity of place cells, by individual rat. The proportions of cells from each rat selective to one (blue), two (green), or all three contexts (red) are shown for the a) 3-week separation, b) 2-week separation control, c) no separation, and d) 2-week separation TMZ groups. Numbers above each rat histogram indicate the total number of cells collected from each rat that exhibited a place field in at least one context.

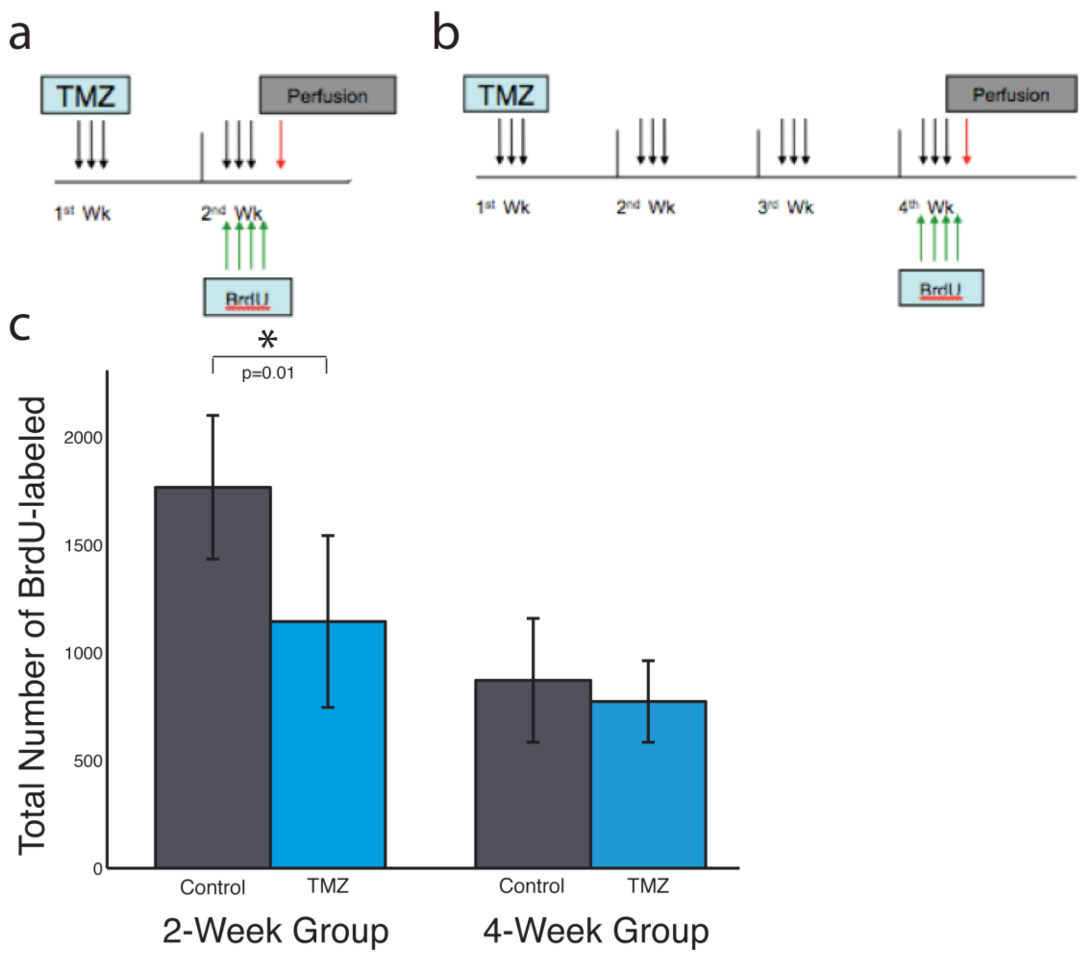

Supplementary Figure S3: The total number of BrdU-labeled cells after 2 and 4 weeks of temozolomide (TMZ) administration. To test the time course of neurogenesis knockdown due to TMZ administration, rats were given either control or 12.5 mg/kg TMZ injections i. p. three times a week for a period of 2 or 4 weeks (**a** and **b**, respectively). To label dividing cells at the end of the 2- or 4-week period, rats were given 50 mg/kg injections of BrdU once a day for 4 days. The mean total numbers of BrdU-labeled cells from each group are shown in **c**. Error bars indicate standard deviation. There was a significant decrease in the total number of BrdU-labeled cells after 2 weeks of TMZ treatment (N = 6 control rats, N = 6 TMZ-treated rats, two-tailed t-test, d.f.= 5, p=0.0065) but no significant difference after 4 weeks of treatment (N = 8 control rats, N=8 TMZ-treated rats).

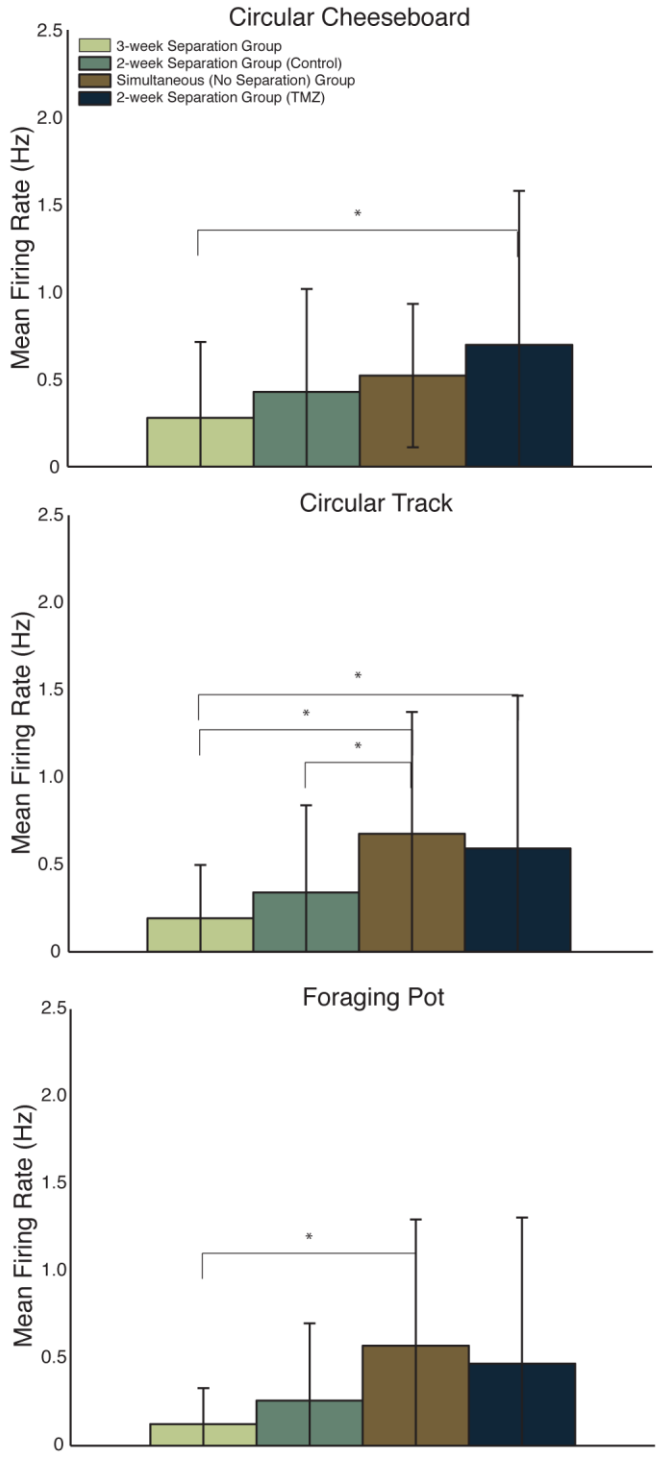

Supplementary Figure S4: The mean firing rate per context across all groups. A two-way ANOVA revealed significant differences in mean firing rate between the 3-week separation

group (N = 72 cells) compared to all other groups as well as between the 2-week separation control group (N = 94 cells) compared to all other groups (d.f.=3, $F=19.59$, $p<0.00001$). There was no significant difference between the 2-week separation TMZ-treated (N = 65 cells) and simultaneous context (N = 52 cells) groups. There was also a main effect due to context, as firing rates were generally lower in the foraging pot than in the other contexts (d.f.=2, $F=3.96$, $p=0.01$). Error bars indicate standard deviation.

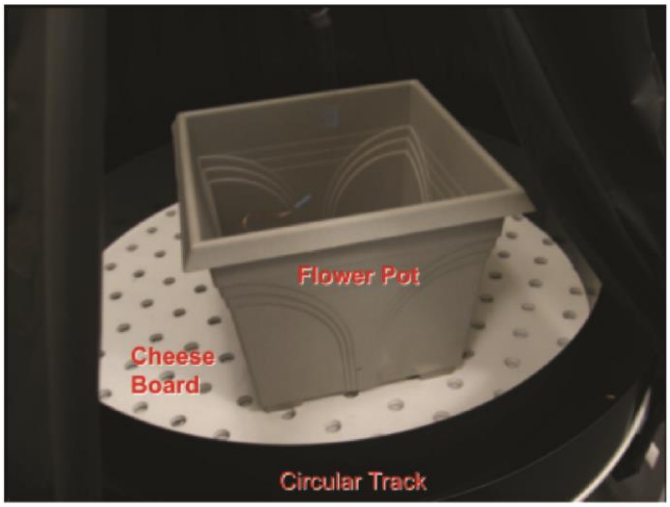

Supplementary Figure S5: Behavioral contexts used in each of the temporal training paradigms. The three behavioral contexts were a 13” x 13” foraging pot containing rat bedding and randomly placed ¼ pieces of Honey Nut Cheerios; a 48” diameter circular track with a food reward in a reliably rewarded location that was shifted to a different track location up to three times per session; a 48” diameter circular cheeseboard with randomly placed food reward and three presentations of a large food reward at random time intervals and random locations that was removed after a period of >30 seconds.

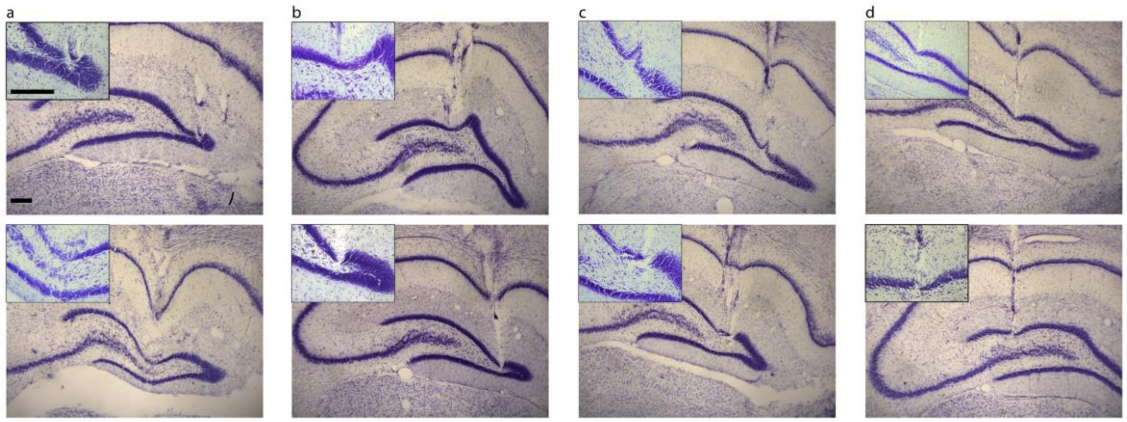

Supplementary Figure S6: Representative electrode track marks from each group. Nissl stained, 40 μm sections from the a) 3-week separation, b) 2-week separation control, c) no separation, and d) 2-week separation TMZ groups at 4x magnification. Insets for each image indicate 20x magnification of the same section. Scale bars indicate 250 μm.

Supplementary Table S1: Context Selectivity as Measured by Firing Rate and Place Field Criteria

	3-week Separation	2-week Separation (Control)	2-week Separation (TMZ)	No Separation
All Granule Cells:	72	94	65	52
Mean Firing Rate > 0.1Hz in:				
One context only:	34 (0.472)	14 (0.163)	3 (0.046)	7 (0.135)
Two contexts:	7 (0.097)	11 (0.128)	6 (0.092)	9 (0.173)
Non-selective:	14 (0.194)	29 (0.337)	35 (0.539)	32 (0.615)
Mean Firing Rate > 0.25Hz in:				
One context only:	27 (0.375)	16 (0.186)	5 (0.077)	8 (0.154)
Two contexts:	4 (0.056)	7 (0.081)	9 (0.139)	8 (0.154)
Non-selective:	5 (0.069)	18 (0.209)	22 (0.339)	22 (0.423)
Demonstrates at least one place field in:				
One context only:	37 (0.661)	27 (0.375)	3 (0.071)	7 (0.149)
Two contexts:	9 (0.161)	19 (0.264)	13 (0.310)	15 (0.319)
Non-selective:	10 (0.178)	26 (0.361)	26 (0.619)	25 (0.532)

A firing rate criterion of 0.1Hz in each context revealed a significant differences between the number of cells selective to one, two, or with activity in all three contexts in comparisons between select groups (*3-week timeline group* v. *2-week timeline control group* chi-square test χ^2= 14.45, d.f.= 2, p= 0.0007, *2-week timeline control group* v. *2-week timeline TMZ-treated group,* χ^2= 8.21, d.f.= 2, p= 0.016). At a firing rate criterion of 0.25Hz, there was a significant difference in context selectivity between the same groups (*3-week timeline group* v. *2-week timeline control group* chi-square test χ^2= 10.7, d.f.= 2, p= 0.005, *2-week timeline control group* v. *2-week timeline TMZ-treated group,* χ^2= 6.11, d.f.= 2, p= 0.047). The number of cells reported in the main text that were selective to one, two, or with activity in all three contexts using a place field criterion are written here for reference.

Supplementary Table S2: Firing Rates, Waveform Statistics, and Spatial Firing Characteristics of Identified Granule Cells

	3-week Separation	2-week Separation (Control)	2-week Separation (TMZ)	No Separation
All Granule Cells:	72	94	65	52
Overall Mean Firing Rate:	0.20Hz ± 0.23	0.35Hz ± 0.44	0.64Hz ± 0.95	0.60Hz ± 0.52
Forage Box:	0.12Hz ± 0.21	0.26Hz ± 0.44	0.47Hz ± 0.84	0.57Hz ± 0.72
Circle Track:	0.20Hz ± 0.32	0.35Hz ± 0.52	0.62Hz ± 0.91	0.71Hz ± 0.73
Cheeseboard:	0.28Hz ± 0.43	0.43Hz ± 0.59	0.70Hz ± 0.88	0.52Hz ± 0.41
Mean Waveform width at half maximum:	182μs ± 81.7	149μs ± 27.8	137μs ± 22.1	155μs ± 40.7
All Identified Place Cells:	56	72	42	47
Median Peak Firing Rate:	5.0 Hz	5.7 Hz	8.0 Hz	7.2 Hz
Number of Fields within a Context:				
Single Field:	44	70	43	47
2 fields:	25	37	20	24
3 fields:	7	16	23	22
4 fields:	6	12	11	9
>4 fields:	3	8	10	10
Median % Coverage of Forage Box:	9.5	21.4	14.6	27.4
Circle Track:	4.7	7.3	10.3	12.3
Cheeseboard:	3.9	5.3	10.3	13.4
Mean Burst Index Score:	0.080 ± 0.059	0.086 ± 0.062	0.080 ± 0.071	0.086 ± 0.058

Where means are indicated, additional values indicate standard deviation.

Supplementary Table S3: Burst Index for Single and Multi- Fielded Cells

	3-week Separation	2-week Separation (Control)	2-week Separation (TMZ)	No Separation
Single Fielded	0.099 ± 0.066<br>N=27	0.105± 0.078<br>N=21	0.032 ± 0.014<br>N=3	0.105 ± 0.060<br>N=11
Multi-fielded	0.071 ± 0.047<br>N=29	0.080 ± 0.051<br>N=51	0.103 ± 0.052<br>N=39	0.086 ± 0.056<br>N=36

Values represent the mean for each group and the standard deviation.

Supplementary Table S4: Number of Fields Within a Context for each Context

	3-week Separation	2-week Separation (Control)	2-week Separation (TMZ)	No Separation
**Cheeseboard**				
Single field:	11	19	5	11
Multiple Fields:	24	40	35	35
**Circle Track**				
Single field:	18	23	10	12
Multiple Fields:	11	31	28	27
**Forage Pot**				
Single field:	15	28	28	24
Multiple Fields:	6	2	1	3

There was a disproportional representation of the three different contexts as indicated by the number of cells exhibiting at least one place field in each. There were no statistically significant changes in these proportions across groups.
